# RETRACTED: De Vecchis et al. Vasopressin Receptor Antagonists for the Correction of Hyponatremia in Chronic Heart Failure: An Underutilized Therapeutic Option in Current Clinical Practice? *J. Clin. Med.* 2016, *5*, 86

**DOI:** 10.3390/jcm13061743

**Published:** 2024-03-18

**Authors:** Renato De Vecchis, Claudio Cantatrione, Damiana Mazzei, Cesare Baldi

**Affiliations:** 1 Cardiology Unit, Presidio Sanitario Intermedio “Elena d’Aosta”, ASL Napoli 1 Centro, via Cagnazzi 29, 80137 Naples, Italy; corneliagracchi58@gmail.com (C.C.); agrippine268@gmail.com (D.M.); 2 Heart Department, Interventional Cardiology, Azienda Ospedaliero-Universitaria “San Giovanni di Dio e Ruggi d’Aragona”, via San Leonardo 1, 84131 Salerno, Italy; cesare.baldi@tiscali.it

The *Journal of Clinical Medicine* retracts the article “Vasopressin Receptor Antagonists for the Correction of Hyponatremia in Chronic Heart Failure: An Underutilized Therapeutic Option in Current Clinical Practice?” [1], cited above.

Following publication, concerns were brought to the attention of the publisher regarding potential redundancy with a previous publication [2] from the same authorship group.

Adhering to our complaint’s procedure, an investigation was conducted by the Editorial Office and the Editorial Board that confirmed a significant overlap between the two publications. As a result, the Editorial Office and Editorial Board consider this publication [1] to be redundant, and therefore have decided to retract it as per MDPI’s retraction policy (https://www.mdpi.com/ethics#_bookmark30) and in line with the Committee on Publication Ethics’ retraction guidelines (https://publicationethics.org/retraction-guidelines).

This retraction was approved by the Editor-in-Chief of the *Journal of Clinical Medicine*.

The authors did not provide a comment on this retraction decision.
